# A comparative study of explainable ensemble learning and logistic regression for predicting in-hospital mortality in the emergency department

**DOI:** 10.1038/s41598-024-54038-4

**Published:** 2024-02-10

**Authors:** Zahra Rahmatinejad, Toktam Dehghani, Benyamin Hoseini, Fatemeh Rahmatinejad, Aynaz Lotfata, Hamidreza Reihani, Saeid Eslami

**Affiliations:** 1 Department of Medical Informatics, Faculty of Medicine, Mashhad University of Medical Sciences, Mashhad, Iran; 2 Toos Institute of Higher Education, Mashhad, Iran; 3 Pharmaceutical Research Center, Pharmaceutical Technology Institute, Mashhad University of Medical Sciences, Mashhad, Iran; 4 Department of Pathology, Microbiology, and Immunology, School of Veterinary Medicine, University of California, Davis, CA USA; 5 Department of Emergency Medicine, Faculty of Medicine, Mashhad University of Medical Sciences, Mashhad, Iran; 6 Department of Medical Informatics, Amsterdam UMC - Location AMC, University of Amsterdam, Amsterdam, The Netherlands

**Keywords:** Machine learning, Prognostic models, Ensemble models, In-hospital mortality, Emergency department, Outcomes research, Computer science

## Abstract

This study addresses the challenges associated with emergency department (ED) overcrowding and emphasizes the need for efficient risk stratification tools to identify high-risk patients for early intervention. While several scoring systems, often based on logistic regression (LR) models, have been proposed to indicate patient illness severity, this study aims to compare the predictive performance of ensemble learning (EL) models with LR for in-hospital mortality in the ED. A cross-sectional single-center study was conducted at the ED of Imam Reza Hospital in northeast Iran from March 2016 to March 2017. The study included adult patients with one to three levels of emergency severity index. EL models using Bagging, AdaBoost, random forests (RF), Stacking and extreme gradient boosting (XGB) algorithms, along with an LR model, were constructed. The training and validation visits from the ED were randomly divided into 80% and 20%, respectively. After training the proposed models using tenfold cross-validation, their predictive performance was evaluated. Model performance was compared using the Brier score (BS), The area under the receiver operating characteristics curve (AUROC), The area and precision–recall curve (AUCPR), Hosmer–Lemeshow (H–L) goodness-of-fit test, precision, sensitivity, accuracy, F1-score, and Matthews correlation coefficient (MCC). The study included 2025 unique patients admitted to the hospital’s ED, with a total percentage of hospital deaths at approximately 19%. In the training group and the validation group, 274 of 1476 (18.6%) and 152 of 728 (20.8%) patients died during hospitalization, respectively. According to the evaluation of the presented framework, EL models, particularly Bagging, predicted in-hospital mortality with the highest AUROC (0.839, CI (0.802–0.875)) and AUCPR = 0.64 comparable in terms of discrimination power with LR (AUROC (0.826, CI (0.787–0.864)) and AUCPR = 0.61). XGB achieved the highest precision (0.83), sensitivity (0.831), accuracy (0.842), F1-score (0.833), and the highest MCC (0.48). Additionally, the most accurate models in the unbalanced dataset belonged to RF with the lowest BS (0.128). Although all studied models overestimate mortality risk and have insufficient calibration (*P* > 0.05), stacking demonstrated relatively good agreement between predicted and actual mortality. EL models are not superior to LR in predicting in-hospital mortality in the ED. Both EL and LR models can be considered as screening tools to identify patients at risk of mortality.

The escalating influx of patients into emergency departments (EDs) has given rise to a critical issue known as emergency overcrowding, resulting in a significant disparity between available resources and the genuine needs of patients^1^. This situation is widely reported and results in a mismatch between scarce resources and the real needs of patients^2^. Effectively addressing this intricate phenomenon necessitates strategic interventions^3,4^. An essential aspect of effective management involves the development of efficient assessment methods to gauge the severity of critically ill patients, predicting outcomes such as deterioration and mortality at the earliest possible stage^5,6^. Employing such risk stratification tools facilitates early detection, intervention, and intensive monitoring of individuals at a heightened risk of morbidity or mortality^7,8^.

Several studies have investigated the application of scoring systems to predict in-hospital mortality, identified by a discharge status of “died” or “died in a medical facility”^6,9–13^. Within the Iranian context, specific studies have utilized scoring systems for predicting in-hospital mortality in the ED, incorporating predictors such as demographic information, vital signs, mechanical ventilation status, oxygen saturation, abnormal electrocardiography findings, and the history of underlying diseases. Notable among these systems are the Acute Physiology and Chronic Health Evaluation (APACHE)^14^, Simplified Acute Physiology Score (SAPS)^14^, and Sequential Organ Failure Assessment (SOFA)^15^. Additionally, an Iranian study compared in-hospital mortality prediction between emergency residents' judgment and prognostic models in the ED, highlighting the superior calibration of mortality risk prediction by SOFA^16^. These investigations collectively underscore the utility of scoring systems in assisting clinicians with timely intervention decisions, crucial for mitigating in-hospital mortality. However, it's noteworthy that existing scoring systems and certain severity indices primarily rely on conventional methods such as logistic regression (LR)^17–21^. These static scores may not fully capture patient progression, necessitating a deeper understanding of how to tailor interventions based on individual patient conditions.

In recent years, significant progress in predictive modeling, particularly through the application of machine learning (ML) methodologies, has significantly enhanced forecasting capabilities across diverse scenarios^22–26^. These cutting-edge approaches have successfully illuminated high-order nonlinear interactions among variables, thereby contributing to more robust predictions^27,28^. Moreover, recent developments in ML models have yielded promising outcomes in predicting clinical scenarios, including mortality within EDs^29–36^. Noteworthy is a study that addressed ML-based early mortality prediction in the ED by quantifying the criticality of ED patients, emphasizing the substantial potential of ML as a clinical decision-support tool to aid physicians in their routine clinical practice^31^. Additionally, another investigation conducted a retrospective comparison between the Modified Early Warning Score (MEWS) and an ML approach in adult non-traumatic ED patients^29^. The study concluded that ensemble stacking ML methods exhibit an enhanced ability to predict in-hospital mortality compared to MEWS, particularly in anticipating delayed mortality.

Ensemble learning (EL), an established ML technique, stands out as a robust approach by amalgamating predictions from multiple models to enhance overall performance and predictive accuracy^37,38^. In the context of predicting in-hospital mortality in emergency medicine, EL models may be a dependable alternative to classical LR-based scoring systems for several reasons: (1) In the domain of emergency medicine, patient outcomes are intricately linked to complex relationships that classical models may struggle to discern; (2) Emergency medicine datasets often exhibit missing information or anomalous values in patient records. Ensemble models exhibit robustness in providing predictions despite encountering such challenges; (3) By combining models that make errors on distinct subsets of the data, ensemble methods contribute to improved prediction accuracy. This diversity proves particularly beneficial in capturing the heterogeneity observed in emergency medicine cases; (4) Ensemble methods demonstrate superior generalization capabilities to new, unseen data. This attribute is crucial in emergency medicine, where patient populations and conditions exhibit variations, demanding a model with robust generalization capabilities; (5) The flexibility in hyperparameter tuning offered by ensemble methods is indispensable when confronted with diverse patient populations and the dynamic nature of evolving medical practices in emergency medicine.

Hence, the present study formulated the hypothesis that EL models might exhibit superior predictive capabilities for in-hospital mortality in EDs compared to traditional LR-based models. While the potential advantages and capabilities of EL techniques in constructing predictive models are acknowledged, the assessment of these models, particularly in comparison to classical LR models, remains limited, especially within the context of Iran. Consequently, the primary objective of this study is to compare the predictive performance of EL models with LR models for in-hospital mortality in EDs within a single-center setting in Iran.

## Material and methods

The current study proposed a framework for comparing the performance of LR and EL models in predicting in-hospital mortality using similar predictors. EL methods included Bagging^39^, Adaboost^40^, Random Forests (RF)^41^, Stacking^42^, and Extreme Gradient Boosting (XGB)^41^. The key challenges associated with in-hospital mortality include mixed data types, a large number of features, unbalanced data, and low performance of developed models in some settings such as EDs, all of which encourage the use of ML models.

**Figure 1:**
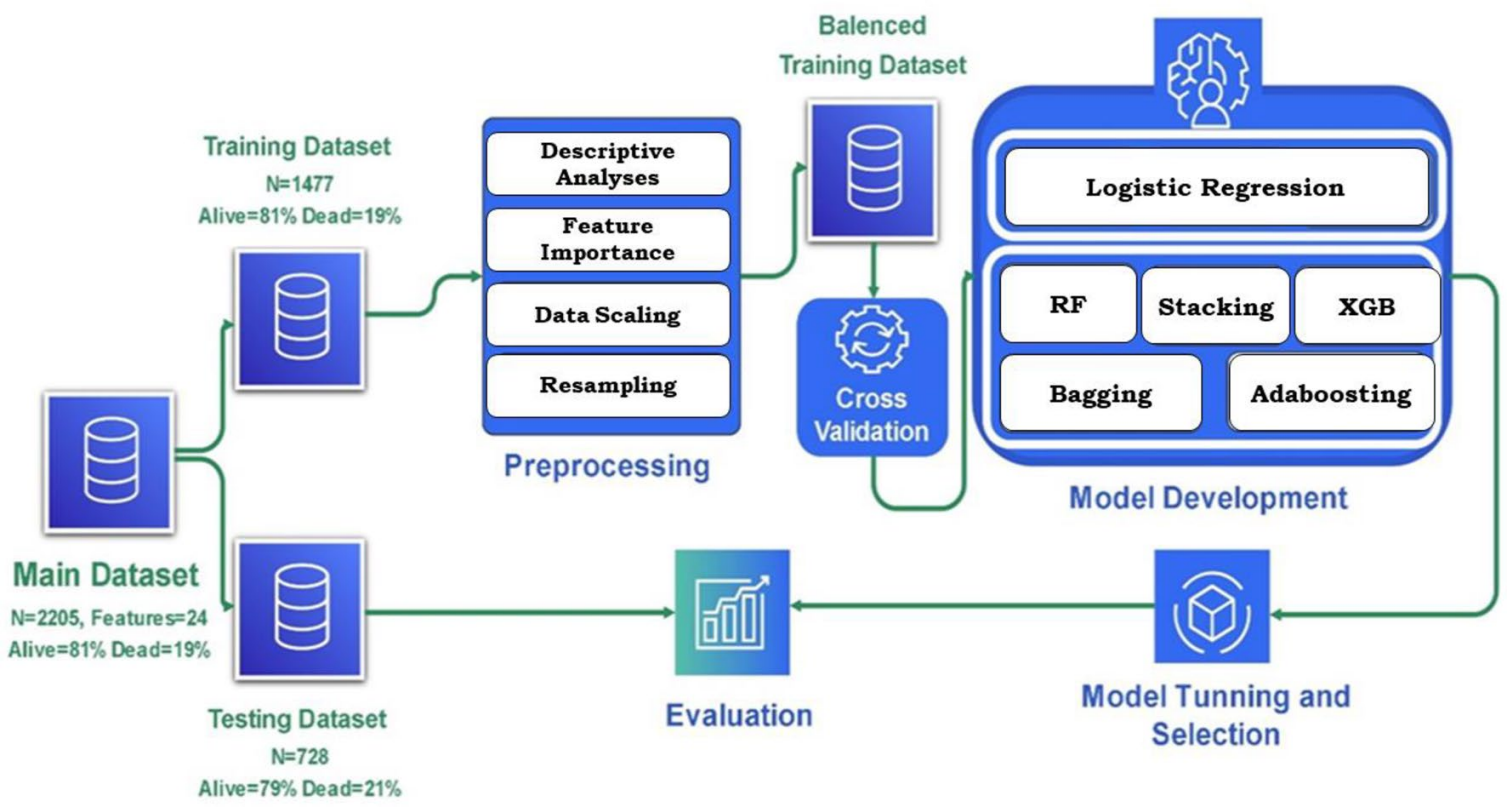
Overview of the proposed ensemble ML models for predicting in-hospital mortality in the emergency department (ED); For the prediction of in-hospital mortality in EDs, logistic regression and five ensemble models were developed and these models were trained and evaluated on the dataset consisting of 2205 patients with 24 predictors, where the number of alive and deceased were 81% and 19%, respectively. This dataset was randomly partitioned into two subsets: the training set includes 67% of data (n = 1477), and the rest of it (n = 728) was assigned to the test set; RF, random forests; XGB, extreme gradient boosting.

To address these challenges, our framework comprises three main phases: pre-processing (Descriptive analysis, Data normalization, and Resampling), model development, and evaluation of the real data set. An overview of the proposed framework is illustrated in Fig. 1**.**

### Study design and dataset description 

This cross-sectional study was conducted in the largest referral ED in the northeast of Iran from March 2016 to March 2017, with over 200,000 patients visiting each year. The study followed the TRIPOD statement for reporting prognostic models, which stands for Transparent Reporting of a multivariable prediction model for Individual Prognosis or Diagnosis. The ethics committee of the Mashhad University of Medical Sciences approved the study (Number: IR.MUMS.MEDICAL.REC.1402.129), and it conformed to the Declaration of Helsinki principles. Informed consent was obtained from all participants or their legal guardian(s) before the study, for experiments involving human participants.

### Inclusion and exclusion criteria

All adult patients, aged ≥ 18 years, with Emergency Severity Index (ESI) triage levels 1 to 3 who presented to the ED throughout the research period were included. Patients triaged directly to the particular department and the intensive care unit (ICU) were excluded from the study. Detailed information about the inclusion and exclusion criteria was presented previously in another report^14^.

### In-hospital mortality as the outcome variable

In this study, in-hospital mortality was defined as an encounter with a discharge status of “died” or “died in a medical facility.” Two classes were defined as the primary outcome: “Alive” and “Deceased,” with their outcomes encoded as binary target value, 0 and 1, respectively.

### Covariates 

The final diagnosis was reported by universal code using the International Classification of Diseases–10^th^ (ICD-10) edition codes. The variables considered in this study are routinely used in traditional scoring systems such as the APACHE and SOFA families for predicting in-hospital mortality or morbidity, which have been previously validated internally in our setting^14,15^. These variables can be categorized into six primary domains: demographic data, vital signs, hematology, biochemistry, Gasometry, and clinical parameters.

The demographic data, such as age and gender, were considered. The vital signs category incorporates parameters such as body temperature (Temp), Mean Arterial Pressure (MAP), including Diastolic Blood Pressure and Systolic Blood Pressure, Respiratory Rate (RR), and the Glasgow Coma Scale (GCS) and pulse. Hematological indicators consist of Hematocrit (HCT), White Blood Cell (WBC) count, and platelet (PLT) count. The biochemistry domain encompasses plasma concentrations of Creatinine (Cr), Potassium (K), Albumin (Alb), Bilirubin (Bil), Sodium (Na), Blood Sugar (BS), pH, and Urea.

Gasometry parameters include Partial pressure of arterial oxygen (PaO_2_), Bicarbonate (HCO_3_), Partial pressure of carbon dioxide (PCO_2_), and Fraction of inspired oxygen (FiO_2_). Lastly, clinical parameters involve the utilization of a Mechanical Ventilator (MV) plus ED status (triage level measured by emergency severity index (ESI), ED arrival method (walk-in vs. ambulance), and exploration of past medical history.

These variables were categorized and participated in model developments as follows:

Continuous predictors: Age, Pulse rate, PaO2, FiO2, GCS, Urine output, RR, Na, BS, pH, Urea, and PLT were considered integer values. However, this difference does not significantly impact the outcome prediction. Both categories receive similar preprocessing steps and thus do not substantially affect predictions. MAP, Temp, HCO3, PCO2, HCT, WBC, Cr, K, Alb, and Bil were used as real values.

Categorical (binary) predictors: MV and Chronic diseases.

### Covariates and outcome variables preprocessing 

In the first phase, to prepare input data for model development, various preprocessing techniques were applied, including descriptive analysis, data normalization, and resampling. The following subsections provide details of these techniques.

#### Step 1: descriptive analyses

As the first step, a descriptive analysis was conducted for both covariates and outcomes. In this analysis, the possible correlations between covariates and outcomes, and their linear relationships, were evaluated using Spearman’s correlation coefficient^43^. Spearman Correlation is a non-parametric test that shares the same assumptions as the Pearson correlation but does not rely on the normality of data distribution.

The Spearman correlation was applied to the continuous covariates, and the significance of their correlations with outcomes was studied based on Confidence Intervals (CIs), R^2^, Bayes Factors (BF10), and power^44^. Moreover, to avoid feature redundancy, the possible pairwise correlation between predictors was examined. Categorical variables were summarized as frequencies and percentages, while continuous variables were expressed as mean ± standard deviation (SD) in both the text and tables.

#### Step 2: scaling and normalization

To mitigate the impact of the varied range of continuous covariates and labels of categorical covariates, data scaling methods were employed. First, for continuous variables, the range of values was transformed using MIN–MAX scaling into the range of [0,1].

#### Step 3: resampling of unbalanced data

A common challenge in mortality datasets is the unbalanced class distribution, which can lead to over-fitting and under-performance of ML models^29^. In the current dataset, the majority class (alive) and the minority class (deceased) represented 81% and 19% of the patients, respectively. To address this issue, a combination of over-sampling and under-sampling techniques, called SMOTETomek, was applied to the training dataset^45,46^. SMOTETomek is a hybrid method that uses under-sampling (Tomek) with an over-sampling (SMOTE) technique. It applies SMOTE for data augmentation on the minority class and Tomek Links (a nearest neighbors’ method) for omitting some of the samples in the majority class. This method can enhance ML models’ performance by making less noisy or ambiguous decision boundaries.

### Model development

In the second phase of our framework, the process of model development was performed, which consisted of (1) determining the best parameters of models using tuning techniques, (2) dividing data into the training and testing datasets using cross-validation, (3) selecting performance measures for the evaluation of models, and finally, (4) developing models and (5) determining the importance of features in the model. The five steps are detailed below.

#### Step 1: tuning of models’ parameters 

One of the main challenges in developing ML models was determining the best parameters. To address this issue, a hyper-parameter tuning technique called GridSearchCV^47^ was carried out. In hyper-parameter tuning, an exhaustive search was performed over the parameters’ space, and as a result, models were optimized based on the best parameters using performance metrics.

#### Step 2: K-fold cross-validation for training and testing 

For the development and evaluation of models, the dataset underwent training and testing phases. The optimal parameters of models were determined using K-fold cross-validation (K-fold)^48^ where the training dataset was divided into K folds, models were trained and validated, and the models with the highest average performance were considered as the optimal ones.

#### Step 3: models’ performance evaluation

To evaluate the ML models, their discrimination power was assessed using performance measures, including Precision, Sensitivity, Accuracy, F-measure (F1), Matthew’s Correlation Coefficient (MCC), Area Under Curve of Receiver Operator Characteristic (AUC-ROC), Area Under Curve of Precision–Recall (AUC-PRC), Calibration Plot, Brier Score (BS), Mean Squared Error (MSE), and the DeLong test^49–54^.

The accuracy metric checks the proportion of correctly classified samples, while F1 is the harmonic mean of precision and sensitivity. The calibration plot illustrates the consistency between predictions and observed outcomes. Comparing the calibration of all models through a scatter plot indicates the amount of agreement between the observed outcomes and predicted risk of mortality.

Moreover, by comparing the models’ performance and their accuracy, the Brier Score is computed, and the DeLong test is performed for pairwise comparison between the AUC-ROC. As Eq. (1) shows, BS is calculated as the mean squared difference between predicted probabilities (P) and actual outcomes (O) for binary classification, providing a comprehensive measure of model accuracy and calibration.

1 $${\text{BS}}=\frac{1 }{{\text{N}}}{\sum }_{{\text{i}}=1}^{{\text{N}}}{\left({{\text{P}}}_{{\text{i}}}-{{\text{O}}}_{{\text{i}}}\right)}^{2}$$

Where, N is the number of observations, P_i_ is the predicted probability for observation i, and O_i_ is the actual outcome for observation i.

The DeLong test is based on the covariance between the models. The test statistic follows a standard normal distribution under the null hypothesis of no difference in AUC between the two models. The significance of the difference is then assessed using the standard normal distribution. Equation (2) shows how the DeLong test statistic is calculated.

2 $${\text{Z}}=\frac{{{\text{AUC}}}_{1}-{{\text{AUC}}}_{2}}{\sqrt{{\text{Var}}\left({{\text{AUC}}}_{1}\right)+{\text{Var}}\left({{\text{AUC}}}_{2}\right)-2\times {\text{Cov}}({{\text{AUC}}}_{1},{{\text{AUC}}}_{2})}}$$

where AUC_1_ and AUC_2_ are the areas under the ROC curves for models 1 and 2, Var(AUC_1_)) and Var(AUC_2_) are their respective variances, and Cov(AUC_1_, AUC_2_) is the covariance between the areas.

This step ensures a robust evaluation of predictive performance and identifies any significant variations. These assessments are vital for enhancing the transparency and reliability of our models, contributing to their validity in predicting in-hospital mortality.

#### Step 4: ML modeling

Our framework included LR^55^ and five ensemble ML methods. EL models are meta-models that develop models by exploiting multiple weak classifiers and integrating obtained results to achieve stronger classifiers or regressors via voting or boosting mechanisms. In this study, EL models, Bagging^56^, AdaBoost^57^, RF^58^, Stacking^42^, and XGB^59^ were applied.

- The Bootstrap AGGregating (Bagging) method is demonstrated using decision tree classifiers. This approach employs bootstrap sampling with replacement to create subsets of the training data. These subsets are then used to independently build weak and homogeneous models. The weak models are trained in parallel, and a more accurate model is produced through the voting method, which generates multiple random subsets from the training dataset and utilizes them to train various Ensemble Learning (EL) models concurrently. Each classification model makes predictions, and their results are averaged to achieve a more robust outcome^39^.
- AdaBoost is a tree-based boosting technique that assigns lower weights to misclassified samples, and these weights are adjusted sequentially during the retraining process. The final classification is achieved by combining all weak models, with the more accurate ones carrying more weight and exerting a greater influence on the final results^60^.
- RF is a robust bagging method that involves creating multiple decision tree models. It addresses two aspects of sampling: reducing the amount of training data and the number of variables. Multiple decision trees are trained on randomly selected training subsets to mitigate overfitting. The final aggregate is derived through a majority voting procedure on the models’ results. Consequently, there is reduced correlation between the models, leading to a more reliable final model^61^.
- Stacked generalization (Stacking) is an ensemble ML model typically comprising heterogeneous models. It generates the final prediction by combining multiple strong models and aggregating their results. In the first level, stacking models consist of several base models (RF, ADA, and GradientBoostingClassifier), while in the second level, a meta-model (LR) is created, taking into account the outputs of the base models as input^42^.
- XGB is a tree-based boosting method that utilizes random sample subsets to create new models, with each successive model aiming to reduce the errors of the previous ones. To mitigate overfitting and reduce time complexity, it employs regularization to penalize complex models, tree pruning, and parallel learning^59^.

More information about the setting of each model is provided in Table 1.

**Table 1:** Parameters of ensemble machine learning models for predicting in-hospital mortality in emergency department.

Models	Parameters
Logistic Regression (LR)	The given configuration includes parameter settings for a model, such as 'C', 'class_weight', 'dual', 'fit_intercept', 'intercept_scaling', 'l1_ratio', 'max_iter', 'multi_class', 'n_jobs', 'penalty', 'random_state', 'solver', 'tol', 'verbose', and 'warm_start' as follows: 'C': 1.0, 'class_weight': None, 'dual': False, 'fit_intercept': True, 'intercept_scaling': 1, 'l1_ratio': None, 'max_iter': 100, 'multi_class': 'auto', 'n_jobs': None, 'penalty': 'l2', 'random_state': None, 'solver': 'lbfgs', 'tol': 0.0001, 'verbose': 0, 'warm_start': False
Random Forest (RF)	The set of parameter configurations for a random forest model, including settings for 'bootstrap', 'ccp_alpha', 'class_weight', 'criterion', 'max_depth', 'max_features', 'max_leaf_nodes', 'max_samples', 'min_impurity_decrease', 'min_samples_leaf', 'min_samples_split', 'min_weight_fraction_leaf', 'n_estimators', 'n_jobs', 'oob_score', 'random_state', 'verbose', and 'warm_start' as follows: 'bootstrap': True, 'ccp_alpha': 0.0, 'class_weight': None, 'criterion': 'entropy', 'max_depth': None, 'max_features': 'auto', 'max_leaf_nodes': None, 'max_samples': None, 'min_impurity_decrease': 0.0, 'min_samples_leaf': 1, 'min_samples_split': 2, 'min_weight_fraction_leaf': 0.0, 'n_estimators': 10,000, 'n_jobs': -1, 'oob_score': False, 'random_state': 1, 'verbose': 0, 'warm_start': False
Bootstrap Aggregating (Bagging)	The set of parameter configurations for a bagging classifier or regressor, including settings for 'base_estimator', 'bootstrap', 'bootstrap_features', 'max_features', 'max_samples', 'n_estimators', 'n_jobs', 'oob_score', 'random_state', 'verbose', and 'warm_start' as follows: 'base_estimator': None, 'bootstrap': True, 'bootstrap_features': False, 'max_features': 1.0, 'max_samples': 1.0, 'n_estimators': 10, 'n_jobs': None, 'oob_score': False, 'random_state': None, 'verbose': 0, 'warm_start': False
AdaBoost	The set of parameter configurations for an AdaBoost classifier, including settings for 'algorithm', 'base_estimator', 'learning_rate', 'n_estimators', and 'random_state' as follows: 'algorithm': 'SAMME.R', 'base_estimator': None, 'learning_rate': 1.0, 'n_estimators': 1000, 'random_state': None
Extreme Gradient Boosting (XGB)	The set of parameter configurations for a gradient boosting classifier or regressor, including settings for 'categorical_features', 'early_stopping', 'l2_regularization', 'learning_rate', 'loss', 'max_bins', 'max_depth', 'max_iter', 'max_leaf_nodes', 'min_samples_leaf', 'monotonic_cst', 'n_iter_no_change', 'random_state', 'scoring', 'tol', 'validation_fraction', 'verbose', and 'warm_start' as follows: 'categorical_features': None, 'early_stopping': 'auto', 'l2_regularization': 0.0, 'learning_rate': 0.01, 'loss': 'auto', 'max_bins': 255, 'max_depth': None, 'max_iter': 100, 'max_leaf_nodes': 20, 'min_samples_leaf': 20, 'monotonic_cst': None, 'n_iter_no_change': 10, 'random_state': 42, 'scoring': 'loss', 'tol': 1e-07, 'validation_fraction': 0.1, 'verbose': 0, 'warm_start': False
Stacking	The provided information contains a comprehensive set of parameter configurations for a stacked ensemble model, including settings for individual estimators such as RandomForestClassifier, HistGradientBoostingClassifier, and AdaBoostClassifier, as well as settings for the final estimator and stack method 'cv': None, 'estimators': [('rfc', RandomForestClassifier(ccp_alpha = 0.1, criterion = 'entropy', n_estimators = 10,000, n_jobs = -1, random_state = 1)), ('xgb', HistGradientBoostingClassifier(learning_rate = 0.01, random_state = 1)), ('ADA', AdaBoostClassifier())], 'final_estimator__categorical_features': None, 'final_estimator__early_stopping': 'auto', 'final_estimator__l2_regularization': 0.0, 'final_estimator__learning_rate': 0.01, 'final_estimator__loss': 'auto', 'final_estimator__max_bins': 255, 'final_estimator__max_depth': None, 'final_estimator__max_iter': 100, 'final_estimator__max_leaf_nodes': 31, 'final_estimator__min_samples_leaf': 20, 'final_estimator__monotonic_cst': None, 'final_estimator__n_iter_no_change': 10, 'final_estimator__random_state': 1, 'final_estimator__scoring': 'loss', 'final_estimator__tol': 1e-07, 'final_estimator__validation_fraction': 0.1, 'final_estimator__verbose': 0, 'final_estimator__warm_start': False, 'final_estimator': HistGradientBoostingClassifier(learning_rate = 0.01, random_state = 1), 'n_jobs': None, 'passthrough': False, 'stack_method': 'auto', 'verbose': 0, 'rfc': RandomForestClassifier(ccp_alpha = 0.1, criterion = 'entropy', n_estimators = 10,000, n_jobs = -1, random_state = 1), 'xgb': HistGradientBoostingClassifier(learning_rate = 0.01, random_state = 1), 'ADA': AdaBoostClassifier(), 'rfc__bootstrap': True, 'rfc__ccp_alpha': 0.1, 'rfc__class_weight': None, 'rfc__criterion': 'entropy', 'rfc__max_depth': None, 'rfc__max_features': 'auto', 'rfc__max_leaf_nodes': None, 'rfc__max_samples': None, 'rfc__min_impurity_decrease': 0.0, 'rfc__min_samples_leaf': 1, 'rfc__min_samples_split': 2, 'rfc__min_weight_fraction_leaf': 0.0, 'rfc__n_estimators': 10,000, 'rfc__n_jobs': -1, 'rfc__oob_score': False, 'rfc__random_state': 1, 'rfc__verbose': 0, 'rfc__warm_start': False, 'xgb__categorical_features': None, 'xgb__early_stopping': 'auto', 'xgb__l2_regularization': 0.0, 'xgb__learning_rate': 0.01, 'xgb__loss': 'auto', 'xgb__max_bins': 255, 'xgb__max_depth': None, 'xgb__max_iter': 100, 'xgb__max_leaf_nodes': 31, 'xgb__min_samples_leaf': 20, 'xgb__monotonic_cst': None, 'xgb__n_iter_no_change': 10, 'xgb__random_state': 1, 'xgb__scoring': 'loss', 'xgb__tol': 1e-07, 'xgb__validation_fraction': 0.1, 'xgb__verbose': 0, 'xgb__warm_start': False, 'ADA__algorithm': 'SAMME.R', 'ADA__base_estimator': None, 'ADA__learning_rate': 1.0, 'ADA__n_estimators': 50, 'ADA__random_state': None

#### Step 5: feature importance 

To indicate the most important covariates in deploying ML models, feature importance was assessed. In this study, SHapely Additive explanations (SHAP) were used to determine the importance of features in the training dataset. This method, based on cooperative game theory, increases the transparency and interpretability of ML models by measuring local and global impacts of features. According to the SHAP values, the most relevant features for the final models were indicated^62^.

In this research, Python 3.9.1 (Anaconda), Scikit-learn, Pandas, and NumPy were used for the development and evaluation of models. Visualization of data and output results were performed using the Matplotlib library. In the following subsections, the developed EL models are evaluated and discussed from four aspects: statistical information, effects of preprocessing (resampling) on data, feature importance in modeling, and comparing results of the models through different viewpoints^59^.

## Results 

### Descriptive analysis results

For predicting in-hospital mortality in EDs, LR and five EL models were developed and evaluated on a dataset comprising 2205 patients with 24 predictors and a binary outcome. The distribution of alive and deceased patients was 1779 (81%) and 426 (19%), respectively. The dataset was randomly split into two subsets: the training set, encompassing 67% of the data (n = 1477), and the test set, with the remaining data (n = 728). In both the training and testing sets, patients were classified into “alive” and “deceased” categories. In the training set, there were 1203 (81%) alive and 274 (19%) deceased patients, while in the testing set, there were 576 (79%) alive and 152 (21%) deceased patients. Despite the almost equal ratio of alive and deceased patients in the initial training and testing sets, all sets were unbalanced in terms of the number of alive and deceased patients.

A total of 2205 patients were included, with a mean age of 61.83 ± 18.49 years, of whom 1169 (53%) were male. Patient ages ranged from 18 to 98 years, with survivors having an age range of 63–77 years and non-survivors in the range of 70–80 years (*P* < 0.001). Baseline characteristics of patients are summarized in Table 2**.**

**Table 2:** Baseline characteristics of population’s study.

Predictors	Overall (N = 2205)	Alive (N = 1779)	Deceased (N = 426)	Train (N = 1477)	Test (N = 728)	*p*-Value
Age	61.83 ± 18.49	60.38 ± 18.77	67.89 ± 15.88	61.81 ± 18.49	61.88 ± 18.49	< 0.001^a^
Pulse	94.46 ± 20.15	93.09 ± 19.53	100.18 ± 21.66	94.47 ± 19.88	94.44 ± 20.71	< 0.001^a^
MAP	93.9 ± 19.02	94.85 ± 18.43	89.92 ± 20.84	94.47 ± 19.09	92.72 ± 18.82	< 0.001^a^
Temp	37.25 ± 0.8	37.26 ± 0.8	37.22 ± 0.83	37.26 ± 0.81	37.22 ± 0.78	0.272^a^
RR	20.26 ± 5.75	19.82 ± 5.24	22.08 ± 7.22	20.2 ± 5.81	20.38 ± 5.62	< 0.001^a^
PaO_2_	93.28 ± 5.61	93.72 ± 5.14	91.45 ± 6.93	93.36 ± 5.37	93.14 ± 6.05	< 0.001^a^
FiO_2_	25.04 ± 10.24	23.98 ± 8.26	29.48 ± 15.29	25.03 ± 10.47	25.06 ± 9.76	< 0.001^a^
HCO_3_	22.14 ± 6.48	22.65 ± 5.94	20 ± 8.03	22.26 ± 6.37	21.89 ± 6.71	< 0.001^a^
PCO_2_	39.14 ± 13.71	39.06 ± 12.44	39.51 ± 18.1	39.22 ± 13.34	38.99 ± 14.44	0.04^a^
GCS	14.42 ± 1.38	14.64 ± 1	13.51 ± 2.15	14.4 ± 1.44	14.46 ± 1.24	< 0.001^a^
HCT	34.76 ± 8.84	34.77 ± 8.76	34.74 ± 9.17	34.67 ± 8.82	34.95 ± 8.89	0.619^a^
WBC	11.9 ± 13.82	11.38 ± 13.92	14.05 ± 13.18	11.83 ± 14.07	12.03 ± 13.3	< 0.001^a^
Cr	2.05 ± 2.38	1.94 ± 2.34	2.5 ± 2.53	2.06 ± 2.43	2.03 ± 2.29	< 0.001^a^
UO	1403.67 ± 308	1428.16 ± 274	1301.41 ± 404	1411.85 ± 305	1387.09 ± 313	< 0.001^a^
Na	136.51 ± 6.68	136.59 ± 6.3	136.15 ± 8.08	136.54 ± 6.64	136.45 ± 6.76	0.008^a^
K	4.34 ± 0.95	4.29 ± 0.88	4.58 ± 1.17	4.33 ± 0.94	4.37 ± 0.97	< 0.001^a^
Alb	3.54 ± 0.59	3.61 ± 0.55	3.27 ± 0.67	3.54 ± 0.58	3.53 ± 0.59	< 0.001^a^
Bili	2.14 ± 4.69	1.84 ± 3.74	3.41 ± 7.31	2.15 ± 4.79	2.12 ± 4.47	< 0.001^a^
BS	151.78 ± 100.85	148.85 ± 97.86	164.01 ± 111.74	150.51 ± 93.88	154.34 ± 113.69	0.014^a^
pH	7.36 ± 0.1	7.37 ± 0.09	7.31 ± 0.14	6.99 ± 0.09	6.99 ± 0.12	< 0.001^a^
Urea	75.55 ± 69.35	66.54 ± 59.68	113.18 ± 90.99	74.24 ± 67.61	78.2 ± 72.72	< 0.001^a^
PLT	222.97 ± 133.2	226.93 ± 130.44	206.45 ± 143.14	220.8 ± 128.73	227.38 ± 141.84	< 0.001^a^
MV	123 (6%)	30 (1%)	93 (4%)	87 (4%)	36 (2%)	< 0.001^b^
CD	433 (20%)	326 (15%)	107 (5%)	286 (13%)	147 (7%)	< 0.001^b^

MAP, mean arterial pressure; Temp, temperature; PaO_2_, partial pressure of arterial oxygen; FiO_2_, fraction of inspired oxygen; HCO_3_, bicarbonate; PCO_2_, partial pressure of carbon dioxide; GCS, Glasgow coma scale; HCT, hematocrit; WBC, white blood cell; Cr, creatinine; Na, sodium; UO, urine output; K, potassium; Alb, albumin; Bili, bilirubin; BS, blood sugar; PLT, platelet; MV, mechanical ventilation; CD, chronic disease.

^a^Analysis by independent-samples t-test; ^b^Analysis by Fisher's exact test.

**Figure 2:**
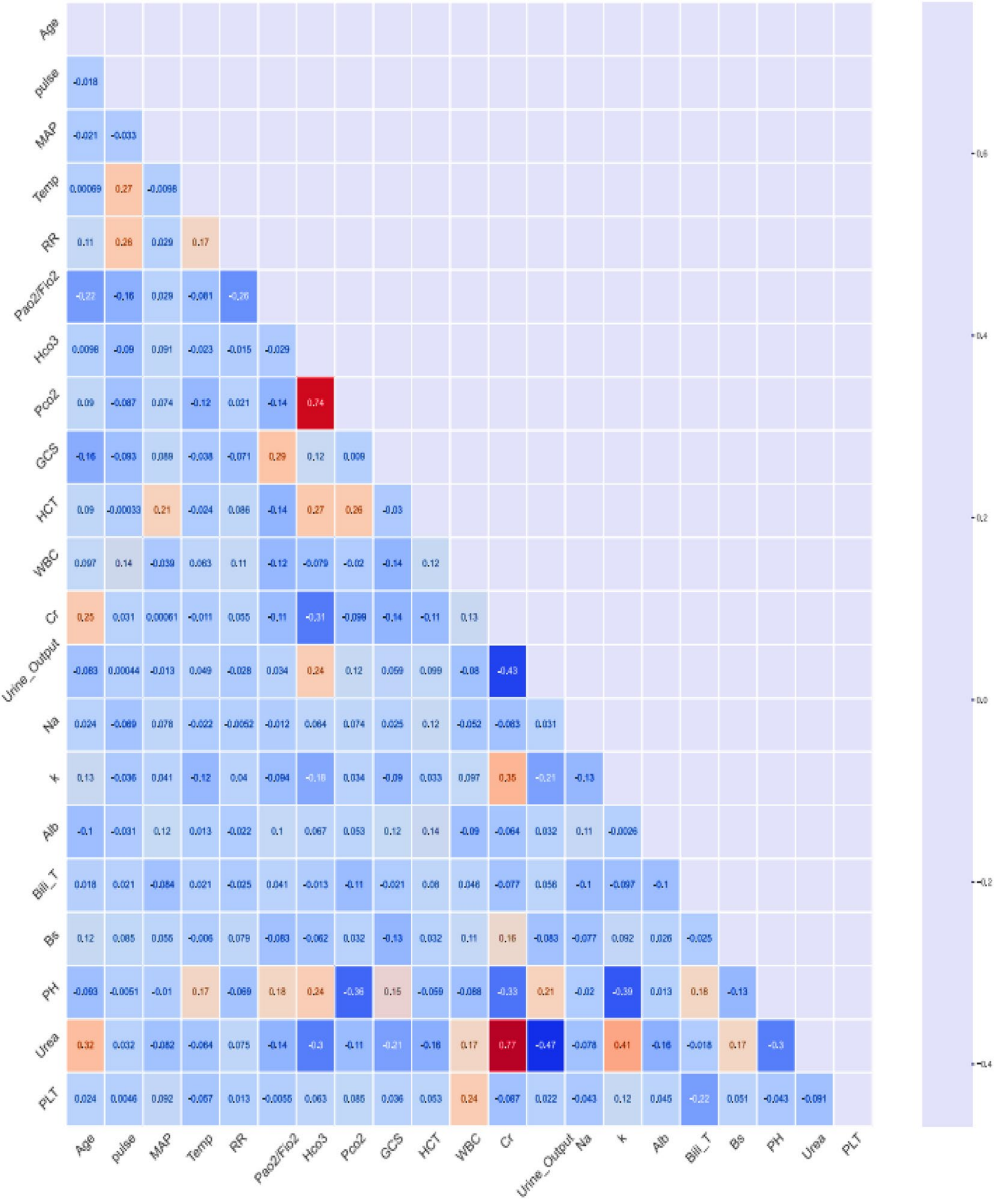
Pairwise correlation coefficient between predictors.

Additionally, the pairwise correlation coefficient between predictors was computed using Spearman Correlation, illustrated in a heatmap plot (Fig. 2). In the heatmap, warm colors indicate high correlation coefficients, while cool ones show low correlation coefficients. This plot indicated that no very strong correlation occurred between continuous predictors with the defined threshold (± 0.8). However, notable correlations, such as high and positive correlations (HCO_3_, PCO_2_: 0.74) and (Urea, Cr: 0.77), as well as moderate and negative correlations (Urine output, Cr: − 0.43) and (Urine output, Urea: − 0.47), were observed.

Moreover, the correlation between covariates and outcomes was assessed, and the results are presented in Table 3, providing correlation coefficients (r), *p*-values, BF10, and statistical power. It is important to note that, while statistically significant correlations were observed for several predictors with the outcome, the magnitude of these correlations is modest. Specifically, only two correlations reached values of 0.35 and 0.22, indicating a generally small effect size.

**Table 3:** Correlation between covariates and outcome.

Covariates	r	95% Confidence intervals		*p*-Value	BF10	Power
		Lower	Upper			
Age	0.16	0.12	0.2	< 0.001	> 100	1
pulse	0.139	0.1	0.18	< 0.001	> 100	1
MAP	− 0.102	− 0.14	− 0.06	< 0.001	> 100	0.998
Temp	− 0.021	− 0.06	0.02	0.324	1/100–1/30	0.167
RR	0.155	0.11	0.2	< 0.001	> 100	1
PaO2	− 0.247	− 0.29	− 0.21	< 0.001	> 100	1
FiO2	− 0.162	− 0.2	− 0.12	< 0.001	> 100	1
HCO3	0.013	− 0.03	0.05	0.539	1/100–1/30	0.094
PCO2	− 0.322	− 0.36	− 0.28	< 0.001	> 100	1
GCS	− 0.001	− 0.04	0.04	0.959	1/100–1/30	0.05
HCT	0.076	0.03	0.12	< 0.001	> 100	0.948
WBC	0.093	0.05	0.13	< 0.001	> 100	0.993
Cr	− 0.162	− 0.2	− 0.12	< 0.001	> 100	1
Urine Output	− 0.026	− 0.07	0.02	0.222	1/100–1/30	0.231
Na	0.12	0.08	0.16	< 0.001	> 100	1
K	− 0.229	− 0.27	− 0.19	< 0.001	> 100	1
Alb	0.133	0.09	0.17	< 0.001	> 100	1
Bili_T	0.059	0.02	0.1	< 0.05	1.305	0.797
BS	− 0.234	− 0.27	− 0.19	< 0.001	> 100	1
pH	0.266	0.23	0.3	< 0.001	> 100	1
Urea	− 0.061	− 0.1	− 0.02	< 0.05	1.555	0.814
PLT	0.347	0.31	0.38	< 0.001	> 100	1
MV	0.068	0.03	0.11	< 0.05	4.067	0.887
Chronic Disease	0.16	0.12	0.2	< 0.001	> 100	1

*BF10, Bayes factor; r, correlation coefficients; MAP, mean arterial pressure; Temp, temperature; PaO_2_, partial pressure of arterial oxygen; FiO_2_, fraction of inspired oxygen; HCO_3_, bicarbonate; PCO_2_, partial pressure of carbon dioxide; GCS, Glasgow coma scale; HCT, hematocrit; WBC, white blood cell; Cr, creatinine; Na, sodium; K, potassium; Alb, albumin; Bili, bilirubin; BS, blood sugar; PLT, platelet; MV, mechanical ventilation.

### Feature importance

To evaluate the importance of each predictor in deploying EL models, we considered the features mentioned in Section “Covariates”, whose correlation with the outcome was analyzed in Table 3. These features in the training dataset were ranked using SHAP^63^, a method widely used for interpreting complex ML models.

**Figure 3:**
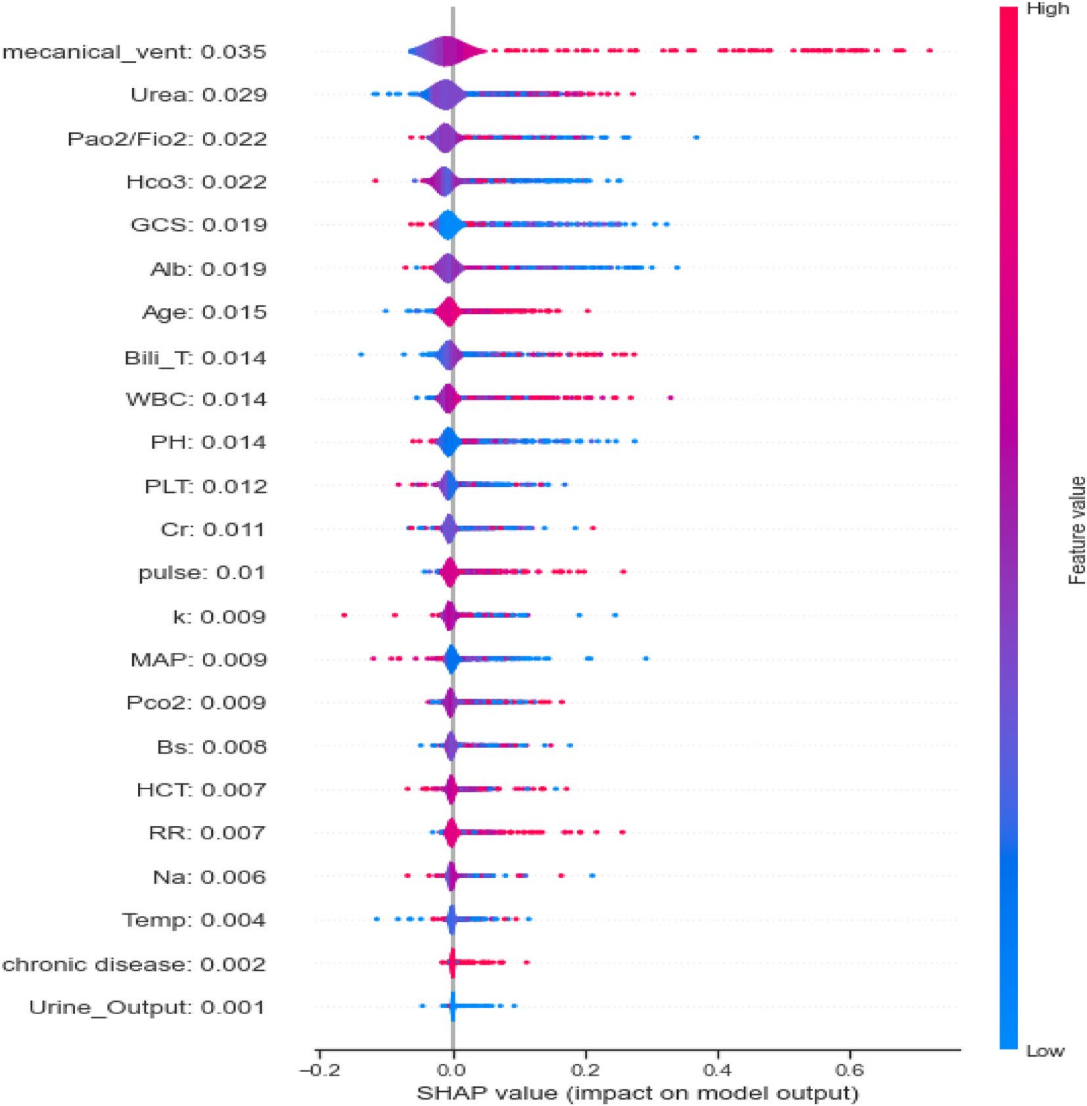
Evaluation of features' importance by SHAP summary plot.

Figure 3 depicts the estimated SHAP values across all samples for the XGB model, demonstrating high performance among EL models. Features are sorted based on SHAP values, with red and blue colors indicating high and low impacts. Additionally, the mean SHAP value for each feature is presented, where higher values indicate higher importance.

According to Fig. 3, predictors such as Urine output, BS, chronic disease, Temp, and Na were considered the least important, while Urea and MV were identified as the most influential factors.

### Resampling effect on data

In the current dataset, the majority class (alive) represented 81% (n = 1779), while the minority class (deceased) was 19% (n = 426). Applying the SMOTE Tomek resampling technique led to a better-balanced training set by increasing the overall number of samples from 1477 to 2402. This resulted in the percentage of the deceased class increasing from 19% (247/1477) to 50% (1201/2402), while the percentage of the alive class reduced from 81% (1203/1477) to 50% (1201/2402) in the training dataset. The study tested the SMOTE Tomek sampling method on a basic LR model, showing improved performance in precision, sensitivity, and F1-measure for the minority class after resampling. Additionally, the resampling method increased the overall AUC-ROC of the LR model from 0.52 to 0.82. As a result, SMOTE Tomek was selected and applied to address the imbalanced data issue in our training data. Table 4 shows the performance comparison of ML Model (LR) before and after resampling.

**Table 4:** Performance comparison of ML model (LR) before and after resampling.

Technique	Class	Precision	Sensitivity	F1-measure
Before resampling	Minority class	**0.70**	**0.30**	**0.42**
	Majority class	0.84	0.97	0.90
After resampling	Minority class	0.84	0.78	0.80
	Majority class	0.80	0.79	0.79

Significant values are in [bold].

ML, machine learning; LR, logistic regression.

### Quality assessment of models

To identify high-performance models, comparisons were made between Logistic Regression (LR) and Ensemble Learning (EL) models (Bagging, AdaBoost, Random Forests, Stacking, and XGB). These models were developed on a training dataset, and their parameters were tuned using GridSearchCV in tenfold cross-validation. The following sections comprehensively evaluate the developed models from three perspectives: (1) predictive performance, (2) discrimination ability, and (3) goodness-of-fit.

### Evaluation of the predictive performance of models

The performance of the models was analyzed based on various measurement metrics. Table 5 demonstrates that among the eight investigated models, ensemble models consistently exhibited the best values across all metrics. For instance, Bagging achieved the highest AUC-ROC (0.84) and AUC-PR (0.64) for predicting in-hospital mortality, while XGB demonstrated superior precision (0.83), sensitivity (0.831), accuracy (0.842), and F1 score (0.833). Additionally, XGB outperformed other models with the highest MCC of 0.48, indicating robust performance in unbalanced data, and RF achieved the lowest BS of 0.128, assessing the calibration of models. Furthermore, a comparison of confusion matrices revealed that XGB, Stacking, and RF had the highest True Negatives (TN) in the range of [0.70, 0.73], while Bagging and LR exhibited the highest True Positives (TP) at 0.15.

**Table 5:** Predictive performance of models on the testing dataset.

Type of Models	Models	AUC-ROC	AUC-PR	Precision	Sen	ACC	F1	MCC	BS	MSE
Classical model	LR	0.826	0.614	0.820	0.779	0.783	0.792	0.440	0.160	0.47
EL models	RF	0.833	0.623	0.817	0.819	0.821	0.819	0.454	**0.128**	0.42
	Bagging	**0.839**	**0.64**	0.827	0.780	0.787	0.800	0.468	0.170	0.47
	Adaboost	0.82	0.61	0.821	0.782	0.782	0.795	0.444	0.250	0.47
	XGB	0.827	0.616	**0.83**	**0.831**	**0.842**	**0.833**	**0.48**	0.136	0.**40**
	Stacking	0.817	0.59	0.812	0.828	0.828	0.813	0.415	0.132	0.41

AUC-ROC, Area Under the Curve of Receiver Operator Characteristic; AUC-PRC, Area Under Curve of Precision–Recall; Sen, Sensitivity; ACC, Accuracy, F1, F-measure; MCC, Matthew’s correlation coefficient; BS, Calibration plot, Brier Score; MSE, Mean Squared Error; EL, Ensemble Learning; LR, Logistic Regression, RF, Random Forests; XGB, Extreme Gradient Boosting.

*Best values in each column are bolded.

### Evaluation of discrimination ability of models 

**Figure 4:**
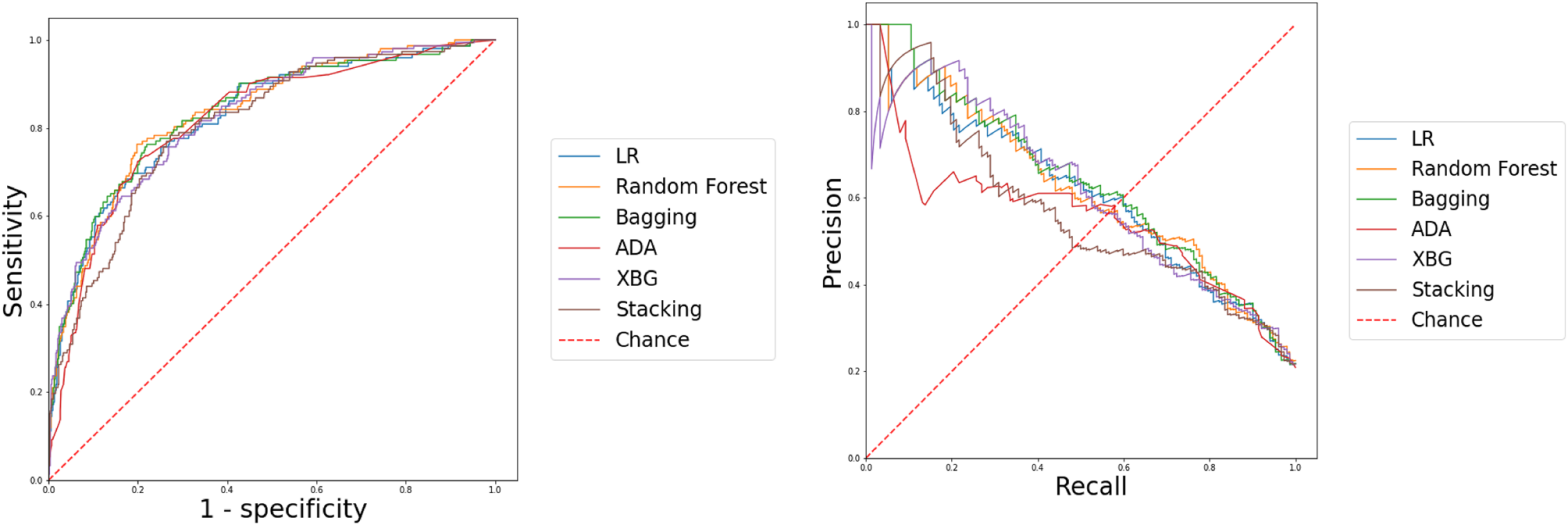
*Left* The receiver operating characteristic curves (AUC-ROC) graphically represent sensitivity versus 1 specificity. *Right* The area under the Precision–Recall curve (AUC-PRC) represents how a model balances the precision and recall.

The pairwise comparison of AUC-ROCs is presented in Table 6, graphically representing sensitivity on the Y-axis and 1-specificity on the X-axis. Additionally, the AUC-PRC is utilized to evaluate how well a model balances precision and recall. In ascending order, Bagging emerged as the most discriminative model with the highest AUROC (0.839, CI 0.802–0.875) and AUCPR = 0.64, followed by RF (0.833, CI 0.797–0.87) and AUCPR = 0.623, XGB (0.826, CI 0.789–0.863) and AUCPR = 0.616, AdaBoost (0.818, CI 0.78–0.857) and AUCPR = 0.61, and Stacking (0.817, CI 0.778–0.856). Figure 4 illustrates that EL models achieved the maximum AUC-PRC, with Bagging leading at 0.64, RF at 0.623, XGB at 0.62, and LR at 0.61.

**Table 6:** Pairwise comparison of AUCs by using the DeLong method.

Delong ROC test	LR	RF	Bagging	Adaboost	XGB	Stacking
LR		0.5114	0.0592	0.5336	0.9849	0.5591
RF			0.6012	0.3365	0.4327	0.2932
Bagging				0.0749	0.3465	0.0859
Adaboost					0.6788	0.961
XGB						0.432
Stacking						

AUC, area under the curve; ROC, receiver operator characteristic; LR, logistic regression; RF, random forests; XGB, extreme gradient boosting.

### Evaluation of goodness-of-fitting in models

**Figure 5:**
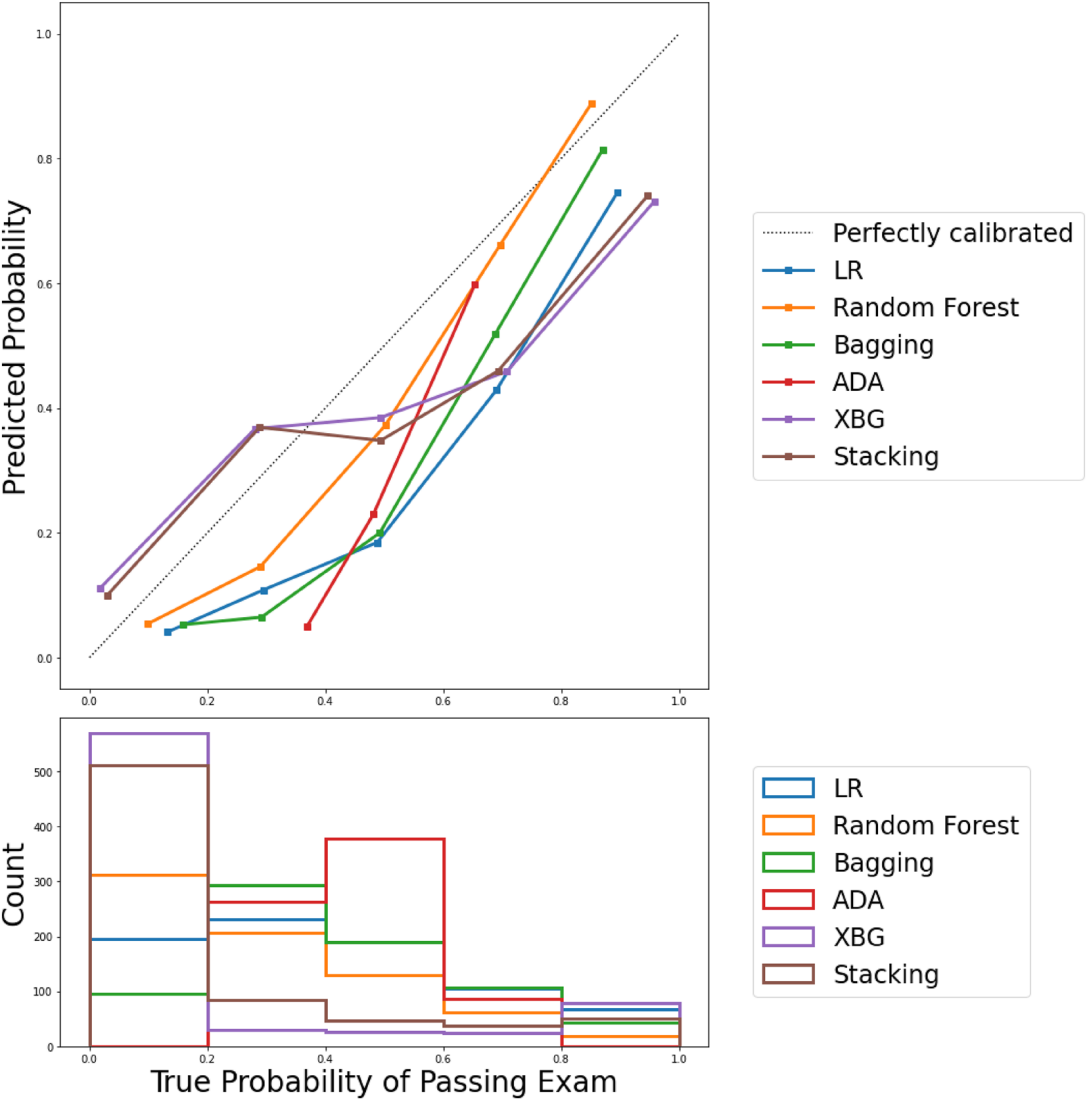
Comparison of models based on calibration plots. A calibration plot is a measure of goodness-of-fit as a graphical presentation of the actual mortality probability versus the predicted mortality probability.

The calibration plot illustrates the consistency between predictions and observations across different percentiles of predicted values, and comparing the calibration of all models through a scatter plot reveals the agreement between predictions and observations. According to Fig. 5, Stacking and RF exhibited greater success in calibration. Moreover, the best BS, a metric comprising calibration and refinement terms, was achieved by RF with a BS of 0.128, followed by Stacking with the lowest BS of 0.132. Conversely, AdaBoost had the highest Brier score at 0.250, indicating a less favorable calibration performance.

## Discussion

The utilization of advanced EL algorithms enables the evaluation of a more extensive range of clinical variables compared to the traditional LR approach. This approach not only allows for the exploration of clinical variables with predictive value but also facilitates the assessment of key features contributing to clinical deterioration. Additionally, EL models offer the potential for automation, eliminating the need for manual review^22^. In preliminary studies, including ours, EL models have proven valuable for clinical decision support, particularly in the stratification of critically ill patients in the ED based on risk factors^64^. Notably, the RF model stands out by providing end-users with the capability to interpret the relative importance of predictive features, enhancing its clinical utility^3^.

### Main findings

The present study applied various ML algorithms to develop models for prognosis patient outcomes based on collected inpatient care data. Our study reports several important findings.

First, when models were trained with both laboratory and clinical data, the highest diagnostic accuracy was achieved. Notably, correlations between (HCO_3_, PCO_2_: 0.74) and (Urea, Cr: 0.77) were observed, showing the strongest correlation, albeit falling just below the defined threshold of 0.8.

Second, utilizing a select set of variables, we found that ensemble methods demonstrated higher performance than classical models such as LR. The LR model's performance remained comparable to high-ranking modern models like RF, Bagging, Adaboost, XGB, and Stacking in predicting in-hospital mortality among ED-admitted patients. No significant differences in discrimination power were observed between the LR and EL models. Regarding overall performance, RF ranked first due to its lowest BS value (0.128). Despite Bagging having the highest discriminatory power among other models, XGB excelled in various metrics, including the highest precision (83%), sensitivity (83.1%), accuracy (84.2%), F1 score (83.3%), MCC (48%), and the lowest MSE (40%).

Third, in pairwise comparisons of AUROC curves, no significant differences were found between XGB and either RF or Bagging, suggesting that XGB performed as well as both.

Lastly, concerning calibration, while all studied models tended to overestimate mortality risk and exhibited insufficient calibration, Stacking demonstrated relatively good agreement between predicted and actual mortality compared to others.

### Comparison to other similar studies

The use of ML models has recently demonstrated effectiveness in predicting outcomes in EDs. For example, ML has been applied to triage in the ED, prediction of cardiac arrest, admission prediction, detection of sepsis and septic shock, identification of patients with suspected infections, and prediction of mortality for sepsis and suspected infections^65^. There is ample evidence consistently suggesting that ML approaches outperform more conventional statistical modeling methods in various contexts, such as ED patients with sepsis^22^, coronary artery disease^66^, and critically ill patients for predicting in-hospital mortality^67^.

In a comprehensive investigation^22^, an RF model was meticulously crafted utilizing an extensive dataset encompassing over 500 clinical variables extracted from electronic health records across four hospitals. Intriguingly, contrary to our findings, this study accentuated the superior performance of this locally derived big data-driven ML approach when compared to both existing clinical decision rules and classical models in predicting in-hospital mortality among ED patients with sepsis. This divergence may be attributed to the substantial scope of the dataset employed. Our study, in contrast, employed 24 variables to construct the ML model. Nevertheless, it is noteworthy that, given the exigent nature of emergency settings with limited time for decision-making, models incorporating fewer predictors may demonstrate enhanced performance and practical utility.

Additionally, another study^29^ utilized an extensive multicenter dataset to develop an EL model for predicting in-hospital mortality among adult non-traumatic ED patients at distinct temporal stages—stratified into intervals of 6, 24, 72, and 168 h. The performance of this model was then compared with that of an LR-based MEWS, calculated using systolic blood pressure, pulse rate, RR, Temp, and level of consciousness. In contrast to our study, this research revealed that EL methods exhibited heightened predictive accuracy for in-hospital mortality, demonstrating notable proficiency in forecasting delayed mortality. It's important to note that our study specifically focused on predicting outcomes at the time of admission, emphasizing prioritization based on the severity of illness. It is recognized that the accuracy of prediction models tends to improve as the temporal proximity to the occurrence of the desired outcome decreases.

Consistent with our investigation, Son et al.^68^ conducted a study in South Korea wherein they examined 21 features spanning vital signs, hematology, Gasometry, and morbidities. Their approach involved the utilization of various ML algorithms and classical models to optimize ML classification models and data-synthesis algorithms for predicting patient mortality in the ED. Notably, their top-performing model employed the Gaussian Copula data synthesis technique in conjunction with the CatBoost classifier, yielding an AUC of 0.9731. Additionally, Adaptive Synthetic Sampling (ADASYN) and SMOTE data-synthesis techniques ensembled by LR resulted in AUCs of 0.9622 and 0.9604, respectively, aligning with our findings. Two additional studies merit attention in the context of our investigation. One study, focusing on sepsis patients admitted to the ED, underscored the importance of variables such as Temp, gasometry, GCS, and the mode of arrival to the ED^69^, all of which align with the parameters considered in our study. The second study concentrated on statistically significant variables, including demographics, vital signs, and chronic illnesses^70^. These parallel investigations emphasize the relevance of these variables in predicting patient outcomes and fortify the comprehensive nature of our study, which incorporates key factors identified in similar research contexts.

Several studies have employed external validation for benchmarking ML and LR methods in various domains, such as the detection of prostate cancer^71^, identification of brain tumors^72^, prediction of in-hospital mortality in patients suffering from ischemic heart disease^73^, and after brain injury^74^. In our study, we validated the model only on the test dataset. Our findings align with those published recently on predicting mortality after traumatic brain injury^75^. The main reason for this concordance might be that ML methods may struggle to effectively analyze non-linear and non-additive signals^37^. Clinical decision-making can be strengthened through interactions with provider intuition, reducing over- and under-triage risks. These models can also help improve resource allocation and operational flow for crisis management teams.

Considering that our models were derived from data encompassing a case-mixed patient population, their applicability is envisaged in analogous settings without a predefined temporal constraint. Nevertheless, we propose the exploration of developing ML models tailored to specific patient groups, such as those afflicted with Sepsis^65^ and Covid-19^5,76,77^, in future research endeavors.

### Strengths and limitations

In this study, we outline both strengths and limitations. Strengths include (i) the analysis of features contributing to model predictions, (ii) the prospective design of the study, which spanned over a year and included a relatively large number of patients, (iii) a systematic comparison of models from different aspects, such as performance, discrimination, and calibration, and (iv) the comparison of classic LR and novel EL approaches.

However, we are aware of several limitations. Firstly, the results stem from a cross-sectional study conducted in a single center. External validation in additional centers is planned for the future based on the findings of this single-center study. Additionally, we limited ourselves to three levels of ESI acuity, making it unclear to what extent these models can be generalized to a broader ED population. Increasing the predictive applicability of models necessitates extended follow-up. Furthermore, clinicians may be hesitant to adopt ML techniques due to their perceived “black box” nature.

Moreover, the features considered in our analysis, such as vital signs, demographic data, and other relevant parameters, primarily exhibit a cross-sectional nature. Consequently, our approach focuses on the initial measurements taken at admission, forming the basis for model generation. We refrain from incorporating temporal features measured at multiple time points to maintain model simplicity and avoid unnecessary complexity. This decision to concentrate on the first measured parameters at admission is deliberate, aiming to strike a balance between model intricacy and practical applicability.

When employing various ML methods, a crucial point for discussion arises: how to reconcile the differences in the sets of features identified by each algorithm. The 24 features under consideration in our study have been internally validated within our setting^14,15^ and are widely recognized as proxies for the performance of vital organs. Consequently, we incorporated all 24 features into the six ML algorithms utilized in our analysis. Given that these features were uniformly included in the ML algorithms, we compared the models’ outputs—namely, the predicted probability of mortality—based on various performance metrics. These metrics indicate that the XGB model outperformed other models across multiple indices.

## Conclusion

In the prediction of in-hospital mortality for patients admitted to the ED, LR demonstrated comparable accuracy to high-ranking EL models. Notably, Bagging exhibited a substantial discrimination power with an AUC-ROC of 0.84, while the optimal overall performance was observed with XGB (Sensitivity = 0.83, Accuracy = 0.83, F1 Score = 0.83, and MCC = 0.48). Furthermore, when compared to LR, XGB demonstrated improvements of 5% in sensitivity, 4% in accuracy, 4% in F1 measures, and 5% in MCC.

The application of these models should prioritize the identification of critically ill patients, particularly in the dynamic and rapidly changing clinical environments of the ED and ICU. This is of utmost importance given the clinical instability of patients in these settings, where conditions evolve rapidly. Future studies are encouraged to explore the development of real-time predictive models, with the integration of these models into electronic health record databases facilitating ongoing evaluation of treatment outcomes. In contrast, conventional scoring systems often necessitate comprehensive and rigid data inputs to yield predetermined outcomes.

## Data Availability

Data analyzed in this study was uploaded to the Harvard Data repository and is available via this link: https://doi.org/10.7910/DVN/7SOYC7.

## Abbreviations

AI: Artificial intelligence
ML: Machine learning
LR: Logistic regression
Bagging: Bootstrap AGGregating
RF: Random forests
ADA: Adaptive boosting
XGB: Extreme gradient boosting
Stacking: Stacked generalization
F1: F-measure
MCC: Matthew’s correlation coefficient
AUC-ROC: Area under curve of receiver operator characteristic
AUC-PRC: Area under curve of precision–recall
BS: Brier score
MSE: Mean squared error
RMSD: Root mean square deviation
R^2^: Coefficient of determination
PaO_2_: Partial pressure of arterial oxygen
FiO2: Fraction of inspired oxygen
GCS: Glasgow coma scale
RR: Respiratory rate
Na: Sodium
BS: Blood sugar
PLT: Platelet
MAP: Mean arterial pressure
Temp: Temperature
HCO_3_: Bicarbonate
PCO_2_: Partial pressure of carbon dioxide
HCT: Hematocrit
WBC: White blood cell
Cr: Plasma creatinine concentration
K: Plasma potassium concentration
Alb: Plasma albumin concentration
Bil: Bilirubin
MV: Mechanical ventilator
